# Long-term insights into who benefits from brood reduction

**DOI:** 10.1093/beheco/araf050

**Published:** 2025-05-30

**Authors:** Hugh Drummond, Cristina Rodríguez, Santiago Ortega

**Affiliations:** Departamento de Ecología Evolutiva, Instituto de Ecología, Universidad Nacional Autónoma de México, 04510, Ciudad de México, México; Departamento de Ecología Evolutiva, Instituto de Ecología, Universidad Nacional Autónoma de México, 04510, Ciudad de México, México; Departamento de Ecología Evolutiva, Instituto de Ecología, Universidad Nacional Autónoma de México, 04510, Ciudad de México, México

**Keywords:** asynchronous hatching, blue-footed booby, brood reduction, life history, maternal survival, sibling conflict

## Abstract

The resource-tracking/facultative brood reduction hypothesis suggests that, under food stress, many altricial birds sacrifice the youngest brood members to enhance the growth and survival of their siblings. Studies examining staggered hatching and food shortage have generally supported this idea, although staggered hatching may serve additional purposes. However, the direct beneficiaries of this selective mortality remain unclear, as most research has overlooked parents and post-fledging outcomes. A life history perspective has rarely been applied to brood reduction. Using a 34-yr individual-based dataset on blue-footed booby (*Sula nebouxii*) reproduction, we investigated whether siblicidal reduction of food-stressed two-chick broods benefits mothers, fathers, or surviving offspring. Results revealed that mothers of reduced broods were ~16% more likely to survive to the next breeding season than mothers of intact broods, indicating a significant maternal benefit. And cessation of sibling competition allowed surviving chicks to achieve fledging body condition, breeding probability, longevity, and lifetime reproductive success comparable to chicks from intact broods. Thus, sacrificing the subordinate chick ensures adequate—not privileged—growth of the surviving sibling and enhances maternal survival, while apparently providing no survival benefit to fathers. However, experimental confirmation is required to validate these findings and further explore the underlying mechanisms.

## Teaser

The brood reduction hypothesis holds that mother birds commonly downsize their broods by allowing the youngest chick to starve, to guarantee sufficient food for the others. But who actually benefits from this infanticide? Some studies have indicated that it can enable remaining chicks to grow better. Now, a study of thousands of blue-footed booby families reveals that infanticide boosts the survival of mothers, but not fathers (who provide much less food).

## Introduction

Lack’s (1947, 1954) facultative brood reduction hypothesis, later generalized and formalized as the resource tracking hypothesis (Temme and Charnov 1987), holds that, in avian species with altricial offspring, parents sometimes forfeit their youngest chicks when food resources are insufficient to support the entire brood. This strategy ensures or bolsters the growth and survival of the remaining offspring. At the outset, mothers produce a large clutch/brood, to maximize fledging success across varying levels of resource availability. To facilitate brood reduction, parents hatch their chicks asynchronously, which establishes a hierarchy among them and promotes starvation-induced mortality through begging competition, unequal food access, and in some species, sibling aggression.

Survivors in reduced broods are expected to grow better, fledge in good condition and recruit into the breeding population with greater probability because per capita provision of food to chicks tends to increase with the smallness of a brood (Nur 1984), and because good body condition facilitates transitioning to independence (eg Krementz et al. 1989) and recruiting into the breeding population (eg Braasch et al. 2009). In a flagship experiment, blackbird (*Turdus merula*) broods were reduced only under poor food conditions and surviving broodmates benefitted from enhanced parental provisioning, increased growth and fledging success, and probable recruitment (Magrath 1989). However, uncertainty over Lack’s hypothesis persists because some studies have contradicted its predictions and other hypotheses offer alternative or complementary explanations of the hatching asynchrony that Lack first sought to explain (reviews in Magrath 1990; Węgrzyn 2023). Moreover, impacts of brood reduction on parents’ and fledglings’ fitnesses are virtually unexplored (Mock and Forbes 1995).

Meanwhile, supplementation of avian broods, which mimics natural brood reduction by reducing feeding demands on parents, has revealed potentially important benefits of reduction to family members, including carryover effects (O’Connor et al. 2014), in the next season. Supplementation of broods has generally improved their growth and their parents´ breeding success (Ruffino et al. 2014), and enabled mountain bluebird mothers (*Sialia currucoides*) to avoid loss of body mass (Garcia et al. 1993). In Ural owls (*Strix uralensis*), supplementation not only enabled parents to reduce their provisioning and mothers to maintain their body mass, but also permitted pairs to nest one week earlier and lay larger clutches in the next season (Brommer et al. 2004). And natural reduction of great tit (*Parus major*) broods seemed to depress the recruitment of surviving nestlings in one population and boost female survival in another (Hõrak Peeter 1995). Hence understanding the adaptiveness of brood reduction may require screening for direct effects on fitness of parents and surviving chicks in the next season.

To analyze the function of facultative brood reduction in a model siblicidal system (Drummond 2019, 2023), we examined the costs and benefits to all members of two-chick families of the blue-footed booby (*Sula nebouxii*). We tested whether parents and their surviving chicks benefit from siblicidal reduction through enhanced post-fledging survival or reproduction. Long-term data from a marked and highly philopatric population allowed us to compare families with two-chick broods that were reduced with families whose broods fledged intact. We compared parents for their survival through the next reproductive season and breeding performance therein; we compared chicks for their body condition at fledging, recruitment success, age at first reproduction, longevity, and lifetime reproductive success.

The most common broods of this long-lived, colony-nesting, socially monogamous marine bird are composed of one or two chicks, although three-chick broods also occur when prey are abundant (Ancona et al. 2011; Bizberg-Barraza et al. 2024). Both parents feed the brood until chicks reach independence at roughly 3 to 4 mo, but mothers, being larger and 27% heavier than fathers, provide roughly three times as much food (Guerra and Drummond 1995). Competition between siblings for food is by begging and violent aggression, and in every brood of two, one sibling becomes dominant, usually the elder, and the other is beaten into submission (Drummond et al. 1986; Drummond and Osorno 1992). The subordinate sibling receives fewer parental regurgitations and grows more slowly but catches up in body mass before fledging (Drummond et al. 1991). However, underfeeding provokes some dominant chicks to increase their daily aggression until the other starves to death or succumbs to the lethal aggression of adult neighbors while seeking adoption (Anderson 1995). A deficit of 20% to 25% in body mass of the dominant chick elicits a 3-5-fold increase in its aggressive pecking, which wins it a ~20% increase in mouth-to-mouth food transfers by parents, to the cost of its sibling (Drummond and Garcia Chavelas 1989). In a minority of broods, it is senior that dies and junior that fledges, implying dominance inversion. Parents never obviously interfere in chick aggression, nor show obvious favoritism when feeding chicks, and it is unclear whether theoretical parent-offspring conflict (Trivers 1974) over brood reduction (O’Connor 1978) has been won by dominant siblings or parents, or resolved by compromise between them (review in Drummond 1987).

## Methods

### Colony monitoring

Between 1989 and 2023, the contents of all booby nests in two study areas of Isla Isabel, off the Pacific coast of Mexico (21°50’59”N, 105°52’54”W), were monitored every 3 d throughout the first three months of every five-month breeding season and every 6 d subsequently (details in Drummond et al. 1986, 2003). Hatchlings were labeled according to hatching order with coloured leg-wires, then numbered plastic bands, and at 70 d (a proxy of fledging, which can occur as early as 80 d) they were weighed, measured (culmen and ulna), and fitted with alphanumeric steel bands. Sex of breeders was identified by voice (females grunt, males whistle) but sex of chicks was normally unknown until they recruited into the breeding population a few years after fledging. Due to limited natal dispersal in this population (30.5 ± 1.7 m in males and 36.6 ± 1.4 m in females; *x* ± *se*) 112 and lifetime philopatry (Kim et al. 2007), this monitoring captured the development and fledging of every clutch and brood, along with the recruitment, annual survival and reproductive performance of offspring of all but the most recent cohorts across their life spans.

### Benefits to parents

We evaluated whether parents that experienced brood reduction (one chick died) outperformed parents of the same sex that successfully fledged both chicks, in terms of survival, early laying, and breeding success in the subsequent year. Our sample was all reproductive events between 1993 and 2022 involving known-age parents (banded at fledging) with a brood of two hatched chicks. First, we compared the probability of surviving to the subsequent breeding season between parents that experienced brood reduction versus those that fledged both chicks. Death of a parent was inferred when an individual failed to breed in the study areas during five consecutive years, and ascribed to the last year it bred (only 2% of breeders take more than five gap years; Drummond et al. unpublished data). Second, for parents that survived, we compared laying dates and breeding success, measured as the number of fledglings produced, in the subsequent year between those that experienced brood reduction and those whose broods fledged intact.

### Benefits to chicks

Using all two-chick broods that hatched between 1989 and 2023, we first compared the fledging body condition (next paragraph) of fledglings from two-chick broods hatched between 1995 and 2023, contrasting individuals of the same hatch order from reduced versus intact broods. We also compared fledglings from reduced versus intact two-chick broods that fledged between 1995 and 2011 for recruitment success (whether they cared for a clutch within the first 12 yr of life), longevity, and lifetime reproductive success (total fledglings produced). Recruitment after 12 yr is rare (Drummond et al. 2011). In addition, for males and females that recruited and died between 1999 and 2017, we compared age at recruitment, longevity, and lifetime reproductive success of individuals of the same sex from intact versus reduced broods.

Body condition at fledging was assessed using a scaled mass index, which adjusts body mass relative to body size, thereby accommodating fluctuations in the body mass-size relationship of each individual over its life span (equation 1; Peig and Green 2009). Senior fledglings from intact and reduced broods weighed 1665 ± 238 grams and 1549 ± 243 grams, respectively, with ulna lengths of 205 ± 11 mm and 205 ± 11 mm, respectively. Junior fledglings from intact and reduced broods weighed 1614 ± 245 grams and 1560 ± 236 grams, respectively, with ulnas measuring 204 ± 11 mm and 203 ± 11 mm, respectively.


$$\begin{array}{l} \;Scaled\;mass\;index\;{\hat{M}}_{i}=M_{i}{\left[\frac{L_{0}}{L_{i}}\right]}^{b} \end{array}$$ (1)

${\hat{M}}_{i}$ denotes the scaled mass index value, $M_{i}$ is body mass (grams), *L*_*0*_ is the population’s mean ulna length (mm), $L_{i}$ is the individual ulna length, and *b* is the scaling exponent, estimated as the slope of the Standardized Major Axis (SMA) regression of the log-transformed body mass on ulna length.

Brood reduction, whether by starvation or attacks by adult neighbors on chicks seeking adoption, respectively, was inferred when monitors found a chick older than 7 d dead on the parents’ territory, or absent from that territory and its surroundings. When both chicks died within nine days of each other (within 3 consecutive nest checks), their deaths were attributed to nest abandonment. Disappearance of chicks younger than 7 d from the nest was attributed to predation by milk snakes (Lampropeltis polyzona), the only local predator of chicks (Ortega et al. 2021). Only chicks younger than 7 d are small enough to be consumed by snakes, given the constraints of their mandibular gape. While sibling aggression begins around 5 to 6 d old (Drummond et al. 1991), brood reduction by expulsion is unlikely at this stage since younger chicks lack the locomotor ability to leave the nest. If, however, siblicide by the older sibling occurred, the chick’s body would have been found in the nest during monitoring and categorized as brood reduction. Lifetime reproductive success was the number of fledglings produced by each male or female breeder over its complete breeding history.

Note that sample sizes in Results differ between parent and chick analyses wherever (1) chicks were excluded because missing morphological or body mass data prevented calculation of their scaled mass index, or (2) parental identities were unknown.

### Statistical analyses

#### Benefit to parents

Models evaluating the benefits of brood reduction to parental mortality (ie the probability of surviving to the next breeding season) and reproductive performance (ie number of fledglings produced) included a two-way interaction between brood reduction (yes, no) and sex (male, female). Both the linear and quadratic expressions of the parent’s age were included as covariables to accommodate quadratic age-related changes in the boobies’ performance (Torres and Velando 2007; Beamonte-Barrientos et al. 2010). Parental identity, nest number, and current year were included as random effects; the first two of these factors accounted for statistical non-independence, while the year accounted for unmeasured environmental variables during the breeding season (eg food availability, parental provisioning, population density; Ancona and Drummond 2013). To measure the influence of brood reduction on the probability of surviving to the subsequent year, we constructed Bernoulli generalized linear mixed models (GLMMs) with logit link function. To analyze laying date and breeding success (number of fledglings produced), we used a LMM with an identity link function and a Poisson GLMM with a log link function.

### Benefit to chicks

Our models assessed differences in life history and reproductive outputs between individuals from intact broods and those from reduced broods. All models incorporated brood reduction and hatching order (senior, junior) as fixed effects, and birth cohort and nest number as random effects. Birth cohort accommodated unmeasured environmental factors during the recruits’ natal years which could affect sibling competition (Ancona and Drummond 2013), while nest number controlled for statistical non-independence when two siblings were included in the same model.

To compare the influence of brood reduction on body condition at fledging and recruitment probability we built two GLMMs. For body condition we employed a Gaussian model with an identity link function, and for recruitment probability we fitted Bernoulli models with a logit link function. In these models we examined a three-way interaction involving brood reduction, hatching order and hatching date (standardized with November 3rd as day 1), the latter to account for intraseasonal weather variations. Inclusion of hatching date was crucial because as the breeding season progresses on Isla Isabel, rainfall decreases, sea surface temperature rises, and primary productivity of the ocean declines, all of which collectively contribute to increased mortality of chicks hatched late in the season (Ortega et al. 2022). The three-way interaction explored whether the possible benefit of brood reduction to the surviving sibling increases with lateness of hatching, as adverse environmental conditions later in the season potentially intensify the antagonistic interactions between competing siblings.

We compared effects of brood reduction on age at first reproduction, longevity and lifetime reproductive success (total number of fledglings produced) of recruits (all of which were sexed) using Poisson models with a log link function. These models examined a three-way interaction involving reduction of the natal brood, sex, and body condition at fledging to test for possible sex-related and long-term effects of nestling growth following brood reduction. The longevity and lifetime reproductive success models included age at first reproduction as a fixed effect. Additionally, lifetime reproductive success was included as a fixed effect on the longevity analysis, while longevity was added as an independent variable to the lifetime reproductive success models. Age at first reproduction was included because late recruitment could reduce the number of breeding years available to a booby; longevity controlled for selective disappearance (van De Pol and Verhulst 2006); and total number of fledglings produced controlled for possible effects of the trade-off between reproduction and somatic maintenance.

All analyses used the R statistical environment (R Development Core Team. 2023). Prior to model fitting, continuous fixed variables were z-score normalized to enhance the interpretability of parameter estimates (Grueber et al. 2011; Cade 2015). To ensure computational stability, prevent overfitting, and mitigate the risk of erroneous estimations of large effect sizes, weakly informative priors were incorporated into all models (Gelman et al. 2008; Lemoine et al. 2016). For all fixed effects in the models, a normal prior of N(0,1) was assigned, implying an expectation that the majority of responses would fall within one standard deviation of the average response values, making large effects an unusual occurrence (Lemoine et al. 2016). Variable standardization was performed using the built-in scale function (R Development Core Team 2023), and the brms function within the brms package was used to fit all models (Bürkner et al. (2024); Stan Modeling Language Users Guide and Reference Manual, 2.35. 2024). For each dependent variable, we calculated the contrast of interest between the estimated mean effects using the emmeans package (Lenth et al. 2022).

The posterior distributions of the parameters, accompanied by their 89% highest posterior density intervals (HPD), representing high-probability interval of parameter values (McElreath 2020), were drawn by executing five randomly initiated Markov chains. Each chain underwent 10,000 iterations, with a burn-in period of 1,000 iterations. Posterior predictive checks were performed for each generalized linear mixed models (GLMMs) using the launch-shinystan function of the shinystan package (Gabry Jonah et al. 2022). Complete model summaries are provided in Supplementary Material. Models that exhibited divergent transitions were excluded to avoid unreliable inferences.

## Results

Among the 4677 two-chick broods observed between 1993 and 2022, 3,327 fledged intact (71%), while 751 (16%) experienced reduction; a further 161 (3%) two-chick broods lost both chicks after an initial reduction, and 435 (9%) were abandoned.

### Benefits to parents

Controlling for age and laying date, mothers of reduced broods were ~16% more likely to survive through the next season than mothers of intact broods, but mothers of reduced broods that nested next season did not lay earlier than or produce more fledglings than mothers of intact broods (Table 1; Table S1-S3). In contrast, fathers with reduced broods performed no better than fathers of intact broods in survival, early laying of their partner or production of nestlings (Table 1; Table S1-S3). Of the 688 male and female breeders that experienced brood reduction and survived through the next season, 75% nested in that season.

**Table 1.** Survival and reproductive performance of mothers and fathers after fledging of their reduced versus intact broods in the previous year.

Fathers	Mean (89% HPD)		Contrast (Δ 89% HPD)	Number of broods	
Parameters	Reduced	Intact	Reduced vs Intact	Reduced	Intact
Survival probability	0.58 [0.19, 0.99]	0.50 [0.07, 0.91]	1.74 [0.70, 2.73]	555	2513
Laying date	105 [94.3, 116]	105 [94.2, 116]	0.00 [-1.46, 1.51]	228	1082
Fledging success	0.69 [0.51, 0.93]	0.78 [0.59, 0.96]	0.89 [0.77, 1.01]	228	1082
**Mothers**	**Mean (89% HPD)**		**Contrast (Δ 89% HPD)**	**Number of broods**	
Parameters	Reduced	Intact	Reduced vs Intact	Reduced	Intact
**Survival probability**	**0.64 [0.26, 0.99]**	**0.47 [0.02, 0.86]**	**3.06 [1.05, 4.90]**	**551**	**2557**
Laying date	105 [94.2, 116]	106 [94.2, 116]	-0.26 [-2.22, 1.68]	256	1095
Fledging success	0.74 [0.56, 0.93]	0.78 [0.59, 0.95]	0.95 [0.83, 1.08]	256	1095

Contrasts (Δ) are expressed as odds ratios (OR), except for laying date, which is expressed as a subtraction between means. OR = 1, no distinguishable difference; OR > 1, positive difference; and OR < 1, negative difference. Contrasts were conclusive (in bold) if their 89% highest posterior density (HPD) values were above or below 1. For laying date, contrasts were deemed conclusive if their HPD intervals did not include zero.

### Benefits to chicks

**Fig. 1.**
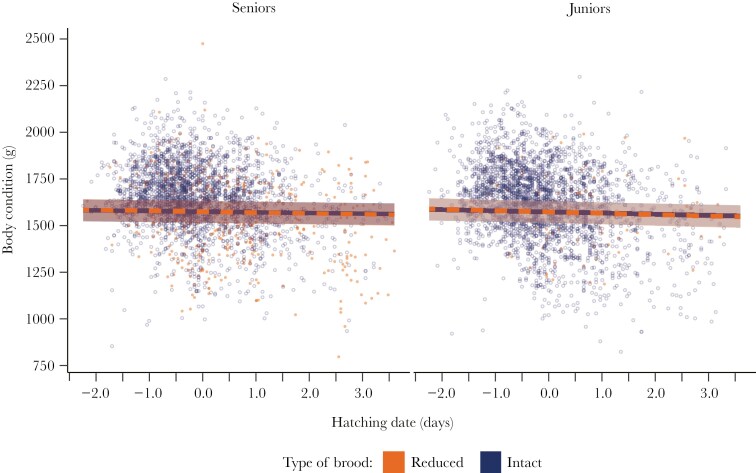
Effect of brood reduction on body condition.

Body condition. For 3381 two-chick broods hatched between 1995 and 2023, body condition at fledging did not differ between members of reduced and intact broods (Fig. 1), nor between seniors and juniors (1574 g and 1573 g, respectively; Table 2 and Table S4). For each one-standard deviation increase in hatching date (~27 d), body condition decreased by about 3.5 g in seniors and juniors.

**Table 2.** Body condition at fledging and recruitment probabilities of seniors and juniors in reduced versus intact broods.

Seniors	Means (89% HPD)		Contrast (Δ 89% HPD)	Number of broods	
Parameters	Reduced	Intact	Reduced vs Intact	Reduced	Intact
Body condition at fledging^1^	1574 [1528, 1621]	1574 [1526, 1619]	-0.12 [-1.74, 1.45]	421	2286
Recruitment probability^2^	0.47 [0.35, 0.60]	0.45 [0.33, 0.56]	1.11 [0.80, 1.40]	255	1633
**Juniors**	**Means (89% HPD)**		**Contrast (Δ 89% HPD)**	**Number of broods**	
Parameters	Reduced	Intact	Reduced vs Intact	Reduced	Intact
Body condition at fledging^1^	1573 [1526, 1619]	1573 [1525, 1618]	-0.09 [-2.29, 2.22]	90	2451
Recruitment probability^2^	0.37 [0.21, 0.53]	0.38 [0.28, 0.49]	1.07 [0.51, 1.58]	50	1632

Contrasts (Δ) are expressed as odds ratios (OR), except for body condition, which is expressed as a subtraction between means. Odds Ratio = 1, no distinguishable difference; OR > 1, positive difference; and OR < 1, negative difference. No contrasts were considered conclusive, as their 89% highest posterior density (HPD) intervals did not exceed or fall below 1, or in the case of body condition, their HPD intervals included zero. The reported means were estimated while accounting for the average values of the ^1^hatching date and ^2^body condition.

**Fig. 2.**
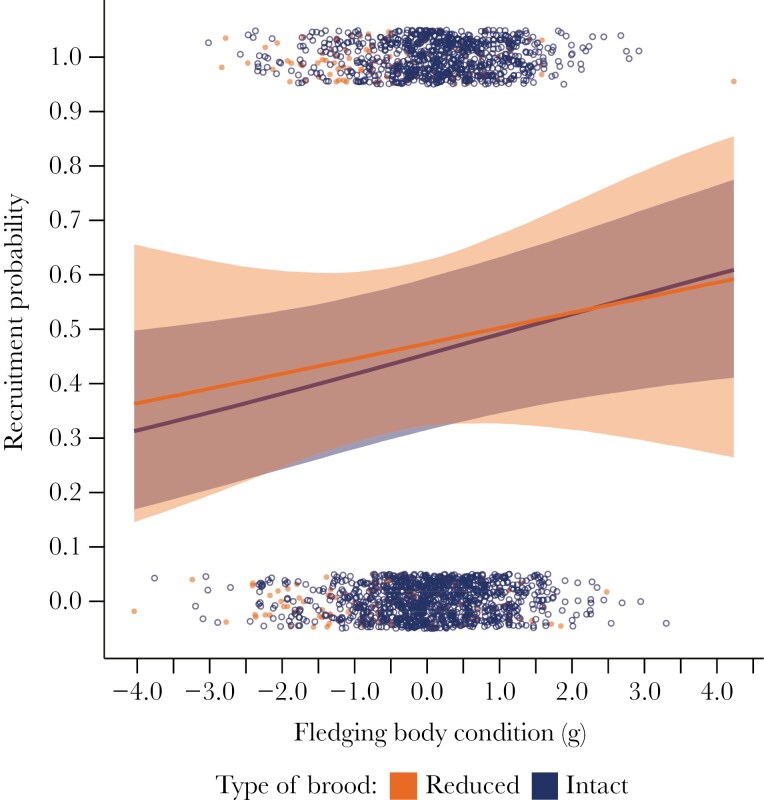
Effect of brood reduction on recruitment probabilities of senior fledglings.

Recruitment. Fledglings from 2347 two-chick broods hatched between 1995 and 2011 provided no evidence that brood reduction improves the survivor’s probability of recruitment (Table 2; Table S5). At the average body condition (~1616 g), seniors from reduced broods were no more likely to recruit than their counterparts in intact broods (47.4% vs 45.4%, respectively), and juniors from reduced broods were no more likely to recruit than their counterparts in intact broods (37.4% vs 38.9%, respectively). On the other hand, recruitment was influenced by both body condition and seniority. Among seniors and juniors, higher body condition at fledging increased the probability of recruitment (β = 0.149 ± 0.067 [89% HPD = 0.042 to 0.256]; slope ± sd) similarly for members of reduced and intact broods (Fig. 2), as did hatching at an earlier date (β = -0.475 [89% HPD = -0.581 to -0.372]). And in broods that fledged intact, seniors were more likely to recruit than juniors (45.4% vs 38.9%, respectively; Table S5), despite the two fledglings having similar body condition (Table 2).

Adult stage. For 767 recruits (404 males and 363 females) that bred and eventually died between 1999 and 2017, reduction (yes/no) of their natal brood was unrelated to their age at first reproduction, longevity, or lifetime reproductive success (Table 3; Tables S6-S8). However, lifetime reproductive success did vary with hatching order, with senior recruits producing approximately 0.2 more fledglings than juniors (Table S8).

**Table 3.** Life history traits and reproductive outputs of recruits from reduced versus intact broods.

Males	Means (89% HPD)		Contrast (Δ 89% HPD)	Number of broods	
Parameters	Reduced	Intact	Reduced vs Intact	Reduced	Intact
Age at first reproduction	4.63 [4.04, 5.22]	5.04 [4.76, 5.32]	0.91 [0.80, 1.03]	51	353
Longevity	7.03 [6.26, 7.81]	7.23 [6.79, 7.67]	0.97 [0.87, 1.07]	51	353
Lifetime reproductive success	1.70 [1.13, 2.25]	1.67 [1.18, 2.13]	1.01 [0.81, 1.21]	51	353
**Females**	**Means (89% HPD**		**Contrast (Δ 89% HPD)**	**Number of broods**	
Parameters	Reduced	Intact	Reduced vs Intact	Reduced	Intact
Age at first reproduction	4.20 [3.63, 4.47]	4.16 [3.92, 4.41]	1.01 [0.87, 1.14]	42	321
Longevity	7.52 [6.74, 8.29]	7.13 [6.71, 7.58]	1.05 [0.95. 1.16]	42	321
Lifetime reproductive success	1.70 [1.13, 2.22]	1.61 [1.15, 2.07]	1.05 [0.86, 1.25]	42	321

HPD = highest posterior density intervals. Contrasts (Δ) are expressed as odd ratios (OR). No contrasts were regarded as conclusive, since their 89% highest posterior density (HPD) intervals neither surpassed nor dropped below 1.

In summary, whether the survivor of a reduced brood was a senior (the usual case) or a junior, loss of its sibling did not enable it to outperform its peer in an intact brood on any measure, from fledging body condition through lifetime reproductive success.

## Discussion

Monitoring over several decades of an insular and highly philopatric population of long-lived blue-footed boobies marked at fledging enabled us to examine from a life history perspective whether facultative, siblicidal reduction of two-chick broods benefits the surviving chick, parents, or both. Fully 71% of broods successfully fledged both chicks, while ~16% experienced reduction, mostly in the first half of the nestling period and predominantly through loss of the junior chick. Another 9% of pairs abandoned their reduced broods within a lapse of 9 d.

### Benefit to parents

Comparisons between reducing and non-reducing families did not reveal earlier laying or greater breeding success by mothers of reduced broods in the next year, but mothers were ~16% more likely to survive to the next breeding season (whether they bred or not) than mothers fledging both offspring. We infer that by roughly halving provisioning of chicks during the remaining weeks of brood care, brood reduction supports mothers’ survival through the next reproductive season, by reducing their provisioning costs. Survival is a key fitness component, positively linked with longevity and lifetime reproductive success, and especially so in a long-lived species (Boyce et al. 2006).

We do not know whether brood reduction was accompanied by reduction in mothers’ foraging and feeding, or improvement in mothers´ body masses. However, while reduction did not enable earlier laying or improved breeding success next season, it increased the probability of surviving through that reproductive season. It remains to be seen whether brood reduction in other avian species with larger broods results in benefit to parents of a similar type and magnitude. Elimination of a single chick from a brood of several may generate a smaller benefit.

In contrast, our monitoring did not detect any direct benefit to fathers from brood reduction even though it might, for all we know, have reduced the cost to them of caring for the current brood. Similarly, when great tit broods (*Parus maj*or) of a rural population underwent natural reduction, it was mothers and not fathers that appeared to cut their costs and benefit from increased survival (Hõrak 1995). Male blue-footed boobies are 28% smaller than females and routinely supply considerably less food to the brood than females except during the first week after hatching (Guerra and Drummond 1995). Brood size manipulations led Velando and Alonso-Alvarez (2003) to infer that blue-footed booby mothers maintain a buffer of nutritional reserves and adjust their food provision to brood size and food needs, allowing greater fluctuation in their body condition than fathers allow in theirs. They suggested that caretaking fathers lack buffer and probably work to a physiological limit or behavioral rule that does not allow body condition to fall below a conservative threshold. Since booby mothers provision more abundantly and flexibly, it is unsurprising that when brood reduction shrinks total food demand of the brood, it is mothers rather than fathers that benefit in their subsequent survival.

### Benefit to chicks

Surviving chicks in reduced broods did not grow heavier than their peers in intact broods, to judge by body condition at fledging. Indeed, there was no evidence that siblicide won the dominant chick any privilege: neither senior nor junior survivors showed higher body condition at fledging or higher probability of recruitment into the breeding population than their peers in intact broods; nor did they, in adulthood, recruit younger, survive longer, or achieve greater lifetime reproductive success than their peers. When it turns out that parents cannot fledge their two offspring in “adequate” body condition, poor personal condition prompts the dominant chick to intensify attacking and kill the subordinate (Drummond et al. 1986; Drummond and Garcia Chavelas 1989), after which the dominant monopolizes parental provision and fledges in adequate condition. Brood reduction directly benefits survivors only by exempting them from the escalating conflict, food deprivation and poor growth they experience while competing for insufficient food with a hungry sibling. That dominant chicks are underweight at the time of brood reduction is known from documentation across five seasons that 1 - 2 d before juniors died their senior sibs’ average weight deficit varied from 21.6% to 25.8% compared to the average senior in the year of fastest growth (Drummond et al. 1986, 1991). Siblicide likely occurs when the parents’ joint provision of food is insufficient to fledge two chicks in good enough condition to eventually recruit while maintaining the mother’s own viability (Drummond and Garcia Chavelas 1989).

We infer the importance of “adequate” condition for fledglings from two observations: the statistically supported positive relationship between fledging condition and recruitment (a few years later), and the absence of differences in mean body condition among seniors, juniors, survivors and survivors´ peers in intact broods, despite large differences among the provisioning and social environments in which they developed.

Brood reduction in this species assures adequate feeding of surviving chicks and also prolongs the lives and reproduction of mothers, two competing functions that are under control of dominant chicks (the executioners) and mothers (the main provisioners). Depleted by roughly 5 mo of continuous breeding effort, food-deprived mothers take advantage of brood reduction to raise their surviving chick in adequate condition and fortify their own viability as breeders in the next season.

In comparison with juniors, senior fledglings were 6.5% more likely to recruit and senior recruits produced 0.2 more fledglings over the life span, despite juniors and seniors not differing in body condition at fledging. This superiority suggests that juniors and seniors are affected over their lifespans by the trained losing versus trained winning (Drummond and Osorno 1992) or slow versus fast early growth (Drummond et al. 1991) that they experience on their natal territories.

O’Connor´s (1978) influential model of the evolution of parent-offspring conflict over avian brood reduction did not consider the possibility of parents benefitting directly from chick deaths, but for comprehensively understanding the adaptiveness of brood reduction, measuring post-fledging effects on parents and chicks is indispensable.

## Conclusions

1. The first study of facultative brood reduction with a life history perspective found that in underfed booby families of two chicks, siblicidal elimination of the subordinate chick by the dominant chick: (a) increases the mother’s survival through next year by ~16% (b) does not directly benefit the father, who provides only a small and fixed amount of parental care; and (c) yields a food bounty that allows the underweight dominant chick to grow adequately, with similar body condition, recruitment, longevity and lifetime reproductive success to chicks in intact well-fed broods. The study is descriptive/correlational, and its causal inferences require confirmation with experimental methods. It also revealed that (d) body condition of fledglings in intact broods does not decline with hatch order, although in this species with aggressive sibling dominance, trained winning and losing, and faster early growth of senior chicks, probability of recruiting does so decline.
2. Fledging of the surviving chick in adequate condition, together with improved post-fledging survival of the mother, are consistent with surviving chicks eating the amount of bounty needed to reach adequate condition and mothers eating the surplus.
3. In studies of avian brood reduction, understanding the assignment of the bounty from nestling deaths among survivors and between survivors and parents requires a life history perspective.
4. Next-generation models of the evolution of brood reduction and associated parent-offspring conflict (cf., O’Connor 1978) should take into account direct effects of reduction on parents and offspring after fledging of the brood.

## Supplementary Material

araf050_suppl_Supplementary_Tables

## Data Availability

Analyses reported in this article can be reproduced using the data provided by Drummond H et al (2025).
