# Supplementary material for: Long-term insights into who benefits from brood reduction
Source: Behav Ecol. 2025 May 30;36(4):araf050. doi: 10.1093/beheco/araf050 (PMC12167230; doi:10.1093/beheco/araf050)
Supplement: araf050_suppl_Supplementary_Tables [file araf050_suppl_supplementary_tables.pdf]

Supplementary material

Table S1. Effect of brood reduction on parental survival.

Parameters	mean	sd	89% HPD	
			Lower	Upper
Intercept	0.503	1.604	-2.020	3.069
Sex* × Brood reduction**				
Female × Intact	-0.538	0.474	-1.284	0.219
Sex*				
Female	0.360	0.480	-0.395	1.128
Brood reduction**				
Intact	-0.472	0.404	-1.103	0.171
Age	0.190	0.176	-0.083	0.470
**Age^2**	**-0.538**	**0.162**	**-0.770**	**-0.295**

Random effects

Parameters	sd	89% HPD		n
		Lower	Upper	
Ring ID	7.225	4.523	9.643	3435
Nest ID	2.137	0.873	3.353	4078
Year	10.501	6.148	14.459	29

Terms whose regression coefficients were statistically different from zero are presented in boldface type. Male* and Reduced** broods were used as reference levels. HPD = highest posterior density.

Table S2. Effect of brood reduction on parental laying date in the following year.

Parameters	mean	sd	89% HPD	
			Lower	Upper
Intercept	105.381	6.881	94.346	116.330
Sex × Brood reduction				
Female × Intact	0.273	0.852	-1.061	1.661
Sex				
Female	0.069	0.832	-1.273	1.377
Brood reduction				
Intact	-0.008	0.930	-1.509	1.465
Age	-0.117	0.692	-1.280	0.934
Age^2	0.488	0.494	-0.305	1.266
Random effects				
Parameters	sd	89% HPD		n
		Lower	Upper	
Ring ID	7.424	2.118	12.293	1558
Nest ID	34.674	33.217	36.085	2068
Year	33.105	25.001	40.836	27
Residual	23.734	22.045	25.482	

Terms whose regression coefficients were statistically different from zero are presented in boldface type. Male* and Reduced** broods were used as reference levels. HPD = highest posterior density.

Table S3. Effect of brood reduction on parental fledging success in the following year.

Parameters	mean	sd	89% HPD	
			Lower	Upper
Intercept	-0.378	0.162	-0.637	-0.122
Sex × Brood reduction				
Female × Intact	-0.071	0.110	-0.247	0.104
Sex				
Female	0.074	0.100	-0.082	0.239
Brood reduction				
Intact	0.118	0.084	-0.013	0.255
Age	0.017	0.029	-0.030	0.062
**Age^2**	**-0.031**	**0.018**	**-0.059**	**-0.001**
Random effects				
Parameters	sd	89% HPD		n
		Lower	Upper	
Ring ID	0.029	0.000	0.059	1558
Nest ID	0.047	0.000	0.094	2068
Year	0.668	0.489	0.842	27

Terms whose regression coefficients were statistically different from zero are presented in boldface type. Male* and Reduced** broods were used as reference levels. HPD = highest posterior density.

Table S4. Effect of brood reduction on fledging body condition.

Parameters	mean	sd	89% HPD Lower	89% HPD Upper
Intercept	1574.046	29.363	1526.278	1619.388
Hatching order* × Hatching date × Brood reduction**				
Junior × Hatching date × reduced	-0.067	0.996	-1.694	1.484
Junior × Reduced	0.028	0.995	-1.525	1.655
Hatching date × Reduced	-0.995	0.986	-2.562	0.573
**Junior** × **Hatching date**	**-2.531**	**0.975**	**-4.083**	**-0.968**
Hatching order*				
Junior	-1.353	0.962	-2.896	0.191
**Hatching date**	**-3.519**	**0.966**	**-5.055**	**-1.969**
Brood reduction**				
Reduced	-0.123	0.996	-1.739	1.452

Random effects

Parameters	sd	89% HPD Lower	89% HPD Upper	n
Birth cohort	140.977	105.876	173.384	24
Nest ID	78.195	71.639	84.559	3384
Residual	143.942	140.124	147.478	-

Terms whose regression coefficients were statistically different from zero are presented in boldface type. Senior* chicks and Intact** broods were used as reference levels. HPD = highest posterior density.

Table S5. Effect of brood reduction on recruitment probability.

Parameters	mean	sd	89% HPD	
			Lower	Upper
Intercept	-0.188	0.292	-0.659	0.266
Hatching order* × Fledging body condition × Brood reduction**				
Junior × Fledging body condition × Reduced	0.369	0.379	-0.254	0.953
Junior × Reduced	-0.158	0.364	-0.721	0.443
Fledging body condition × Reduced	-0.036	0.150	-0.274	0.204
Junior × Fledging body condition	-0.087	0.082	-0.218	0.045
**Fledging body condition**	**0.149**	**0.067**	**0.042**	**0.256**
**Hatching order***				
**Junior**	**-0.271**	**0.080**	**-0.399**	**-0.144**
**Hatching date**	**-0.476**	**0.066**	**-0.581**	**-0.372**
Brood reduction**				
Reduced	0.082	0.170	-0.186	0.358

Random effects

Parameters	sd	89% HPD		n
		Lower	Upper	
Birth cohort	0.960	0.603	1.304	14
Nest ID	0.489	0.177	0.772	2341

Terms whose regression coefficients were statistically different from zero are presented in boldface type. Senior* chicks and Intact** broods were used as reference levels. HPD = highest posterior density.

Table S6. Effect of brood reduction on age at first reproduction

Parameters	mean	sd	89% HPD	
			Lower	Upper
Intercept	1.598	0.038	1.538	1.659
Sex* × Fledging body condition × Brood reduction***				
Female × Fledging body condition × Reduced	-0.054	0.114	-0.234	0.130
Female × Reduced	0.094	0.113	-0.090	0.272
Fledging body condition × Reduced	-0.014	0.073	-0.129	0.102
Female × Fledging body condition	0.001	0.037	-0.058	0.061
Fledging body condition	-0.002	0.026	-0.044	0.041
Sex*				
**Female**	**-0.191**	**0.036**	**-0.250**	**-0.134**
Hatching order**				
Junior	0.037	0.035	-0.021	0.093
Brood reduction***				
Reduced	-0.088	0.081	-0.217	0.043

Random effects

Parameters	sd	89% HPD		n
		Lower	Upper	
Nest ID	0.022	0.000	0.045	685
Birth cohort	0.068	0.0009	0.115	13

Terms whose regression coefficients were statistically different from zero are presented in boldface type. Males*, Seniors**, and Intact*** broods were used as reference levels. HPD = highest posterior density.

Table S7. Effect of brood reduction on longevity.

Parameters	mean	sd	89% HPD Lower	89% HPD Upper
Intercept	1.968	0.041	1.906	2.032
Sex* × Fledging body condition × Brood reduction***				
Female × Fledging body condition × Reduced	-0.063	0.082	-0.194	0.068
Female × Reduced	0.080	0.083	-0.053	0.213
Fledging body condition × Reduced	-0.016	0.056	-0.106	0.072
Female × Fledging body condition	0.030	0.028	-0.016	0.075
Fledging body condition	-0.004	0.021	-0.039	0.029
Sex*				
Female	-0.013	0.029	-0.060	0.032
**Age at first reproduction**	**0.173**	**0.013**	**0.152**	**0.195**
**Lifetime reproductive success**	**0.279**	**0.014**	**0.257**	**0.302**
Hatching order**				
Junior	0.018	0.028	-0.026	0.062
Brood reduction***				
Reduced	-0.029	0.065	-0.132	0.076

Random effects

Parameters	sd	89% HPD Lower	89% HPD Upper	n
Nest ID	0.017	0.000	0.034	685
Birth cohort	0.094	0.030	0.153	13

Terms whose regression coefficients were statistically different from zero are presented in boldface type. Males*, Seniors**, and Intact*** broods were used as reference levels. HPD = highest posterior density.

Table S8. Effect of brood reduction on lifetime reproductive success.

Parameters	mean	sd	89% HPD	
			Lower	Upper
**Intercept**	**0.555**	**0.185**	**0.259**	**0.843**
Sex* × Fledging body condition × Brood reduction***	0.146	0.147	-0.094	0.374
Female × Fledging body condition × Reduced				
Female × Reduced	0.035	0.153	-0.208	0.279
Fledging body condition × Reduced	-0.008	0.106	-0.176	0.160
Female × Fledging body condition	-0.075	0.056	-0.164	0.014
Fledging body condition	0.048	0.045	-0.024	0.119
Sex*				
Female	-0.036	0.060	-0.133	0.059
**Age at first reproduction**	**-0.352**	**0.032**	**-0.403**	**-0.300**
**Longevity**	**0.636**	**0.030**	**0.587**	**0.684**
**Hatching order****				
**Junior**	**-0.114**	**0.053**	**-0.199**	**-0.027**
Brood reduction***				
Reduced	0.011	0.124	-0.182	0.213

Random effects

Parameters	sd	89% HPD		n
		Lower	Upper	
Nest ID	0.294	0.227	0.360	685.000
Birth cohort	0.576	0.338	0.798	13

Terms whose regression coefficients were statistically different from zero are presented in boldface type. Males*, Seniors**, and Intact*** broods were used as reference levels. HPD = highest posterior density.
